# Rcor2 Is Required for Somatic Differentiation and Represses Germline Cell Fate

**DOI:** 10.1155/2022/5283615

**Published:** 2022-03-19

**Authors:** Lijuan Pei, Hongjie Zhang, Meihui Zhang, Yixuan Wang, Ke Wei

**Affiliations:** Institute for Regenerative Medicine, Shanghai East Hospital, Shanghai Institute of Stem Cell Research and Clinical Translation, Shanghai Key Laboratory of Signaling and Disease Research, Frontier Science Center for Stem Cell Research, School of Life Sciences and Technology, Tongji University, Shanghai 200092, China

## Abstract

Rcor2, the corepressor 2 of REST, a transcriptional repressor, is predominantly expressed in embryonic stem cells (ESCs) and plays a major role in regulating ESC pluripotency and neurogenesis. The function of Rcor2 in development of other germ layers is yet unclear. We utilized a *Rcor2^−/−^* mouse embryonic stem cell (mESC) line to investigate the role of Rcor2 in mESC differentiation. *Rcor2^−/−^* mESC shows reduced proliferation and severely compromised capacity to differentiate to all three germ layers. In contrast, *Rcor2* knockout promotes primordial germ cells (PGCs) specific gene expression and possibly PGC formation. Mechanistically, we revealed that Rcor2 inhibits expression of genes required for PGC development, such as *Dppa3* and *Dazl*, by associating to their promoters and enhancing local suppressive H3K9me3 modifications. Our results suggest that Rcor2 plays an important role in somatic cell fate determination by suppressing PGC differentiation through regulating epigenetic modifications of PGC specific genes.

## 1. Introduction

The REST (the RE-1 silencing transcription factor) is a transcription factor known to suppress gene expression in multiple cell types [1]. Various studies have showed that the REST regulatory network controls the transcription of genes important for self-renewal and pluripotency in embryonic stem cells (ESCs) [2]. However, REST itself is not directly required for pluripotency [2]. The transcription silencing effect of REST is dependent on CoRESTs, including Rcor1, Rcor2, and Rcor3, as its transcriptional corepressors [3]. CoRESTs play an important role in neuron differentiation during brain development [4] and modulate mouse embryonic stem cell differentiation [5]. LSD1 (lysine demethylase 1A) is a histone demethylase, which regulates the expression of key developmental regulators [6]. CoRESTs are also required for the demethylation function of LSD1 by forming the LSD1/CoREST/HDAC complex to regulate expression of genes during early embryonic development [7].

Multiple studies showed that Rcor2 is a subunit of LSD1 complex that regulate neurogenesis [8, 9]. In addition, Rcor2 has been shown to be predominantly expressed in ESCs, interacting with LSD1 to regulate ESC pluripotency, as knocking down *Rcor2* severely impaired pluripotent gene expression in ESCs [10]. Recently, Rcor2 was identified in the protein complex formed at the Nanog promoter in ESCs [11]. Further studies confirmed that Rcor2 is a component of the pluripotency regulatory network and is fine-tuning of pluripotency genes and is required for efficient ESC differentiation [11]. However, the specific functions of Rcor2 in early development of lineages other than ectodermal and neural fates are not well understood.

Here, we utilized a *Rcor2^−/−^* mouse ESC (mESC) line as an *in vitro* model to investigate the role of Rcor2 in differentiation of all lineages, providing a systemic overview of Rcor2's function in early cell fate determination during differentiation.

## 2. Materials and Methods

### 2.1. Animals

All experiments involving animals were conducted in accordance with the Tongji University Guide for the use of laboratory animals and the National Institutes of Health Guide for the Use and Care of Laboratory Animals (8th Edition, 2011) and were approved by the Institutional Animal Care and Use Committee (IACUC) of Tongji University. The mice have caged 12 hours light/dark cycles and given water and food and monitored daily for health. *Rcor2^−/−^* mice (C57BL/6N-Rcor2tm1a (EUCOMM) Wtsi) were obtained from the Welcome Trust Sanger Institute Mouse Genetics Projects (Sanger MGP). *Rcor2^Flag^* mice were generated by knocking in 3 × Flag sequences adjacent to the start codon ATG of *Rcor2* gene locus by CRISPR/Cas9 as previously reported [8].

### 2.2. mESC Culture

For ESC derivation, *Rcor2^−/−^* and the parental WT blastocysts and *Rcor2^Flag^* blastocysts were flushed from the uterus of plugged female mice at day 3.5 and then transferred onto mitomycin C-treated mouse embryonic fibroblasts (MEFs) in ES derivation medium containing knockout DMEM, 15% Knockout Serum Replacement (KSR), 1 mM L-glutamine, 0.1 mM mercaptoethanol, 1% nonessential amino acid, and 1000 U/ml LIF for 5-7 days before passaging.

mESCs were maintained in 2i medium, composed of DMEM (Gibco, 11965092) containing 4.5 g/l glucose medium (Invitrogen, 41965-039), supplemented with 2 mM L-glutamine (Invitrogen, 25030081), 0.1 mM nonessential amino acids (Invitrogen, 11140-035), 0.1 mM 2-mercaptoethanol (Sigma, M-7522), 1 mM sodium pyruvate (Invitrogen, 11360), PD0325901 (1 *μ*M, Selleck, S1036), CHIR99021 (3 *μ*M, Selleck, S1263), 1000 U/ml LIF (Milipore, 3192006), and 15% FBS on at 37°C in a 5% CO_2_ humidified chamber. Cells were cultured on 0.3% gelatin-coated dish.

Genotyping of the *Rcor2^−/−^* mESC was carried out by PCR on genomic DNA with the following primers:

Forward: GCCAACTTCACTCCCTTCCCT (exon5)

Reverse: GCTTGGGGTCTCCAGTATCCG (exon7)

Wild-type cells yield a 503 bp PCR product while no PCR product can be detected from *Rcor2^−/−^* cells.

### 2.3. mESC Spontaneous Random Differentiation

ESCs cultured in 2i medium for 2 days and ES medium (subtracting 2i from 2i medium) for 1 day. mESCs were dispersed into single cell with 0.05% trypsin at day 0 and cultured in the differentiation medium (IMDM (Gibco, 12200036) which contains 10% FBS, 4.5 × 10^−4^ M monothioglycerol (Sigma, M1753), 2 mM L-glutamine, and 0.5 mM ascorbic acid (Sigma, A4544)) in the petri dish in suspension culture as embryoid body (EB) at 50∗10^4^ cells per dish. Differentiation medium was changed at day 2; EB were resuspended in differentiation medium and plated into two 10 cm dish (gelatin coated) at day 4, then change medium every 2 days.

### 2.4. Cardiomyocyte Differentiation from mESCs

mESCs were cultured in the differentiation medium, and EB were resuspended in differentiation medium, added 2 *μ*M Dorsomorphin (Selleck, S7306), and plated two 10 cm dish (gelatin coated) at day 4, then change medium every 2 days.

### 2.5. Quantitative Real-Time PCR (qRT-PCR)

The RNA was extracted using TRIzol extraction reagent (Invitrogen, 15596018). The complementary DNA was synthesized using PrimeScript™ RT reagent Kit (Takara, RR047A). RT-PCR was performed using SYBR Green Premix Ex Taq (Takara, AK8806) in a CFX384 Real-Time Systems (Bio-Rad, C1000, Touch). Sequences of the primers used are shown in Supplementary Table 1.

### 2.6. Western Blot

Western blot experiments were performed following the protocol provided by the Bio-Rad website, and the blots were imaged by ChemiScope 600 EXp (CLinX).

Primary antibodies are as follows: Rcor2 (Novus, NBP1-92323), H3K9me3 antibody (abcam, ab8898), and H3K4me1 (CST, 5326).

### 2.7. RNA-Seq

Bulk RNA-sequencing was performed at Novogene (Beijing, China) using the Illumina HiSeq 2500 instrument. Raw reads were aligned to the mm10 reference genome using the HISAT2 software with default paired-end settings. Transcripts were assembled using the StringTie, after being sorted by the SAMtools. Differential expression analysis was performed by the edgeR R package.

### 2.8. Immunofluorescence Staining

Cells from all groups were fixed for 1 hour in 4% PFA in room temperature and then stained for DAPI (Mpbio, 157574), *α*-actinin (abcam, ab5694), and Stella (abcam, ab19878). Images were acquired by fluorescence microscopy (Zeiss, Axio Vert A1).

### 2.9. Flow Cytometry

Cells were fixed with fixation/permeabilization solution (BD, 554174) at 4°C for 30 minutes, then collected by centrifugation, washed in 1x BD perm/wash buffer, incubated with Stella antibody at 4°C for 1.5 h and secondary antibody at 4°C for 1 h, filtered through a 40 *μ*m filter screen, and immediately analyzed on a flow cytometer (BD FACSVerse). 10,000 events were collected and gated through doublet discrimination for analysis.

### 2.10. ChIP–qPCR

EB at day 2 of differentiation were collected and cross-linked with Dynabeads (Invitrogen 10002D) coupled with Flag M2 antibody (Sigma, F1804) and H3K9me3 antibody (abcam, ab8898) at 4°C overnight. Followed by reverse-crosslink and DNA purification with Maxtract kit (Qiagen 129046), eluted DNA was used for qPCR analysis with input DNA as control.

### 2.11. Statistical Statistics

We use the student's *t*-test to determine the statistical significance of data comparisons. All variables were expressed as mean ± SEM. ∗ means *p* < 0.05, ∗∗ means *p* < 0.01, and ∗∗∗ means *p* < 0.001.

## 3. Results

### 3.1. *Rcor2* Is Required for Mouse Embryonic Stem Cell Proliferation

**Figure 1:**
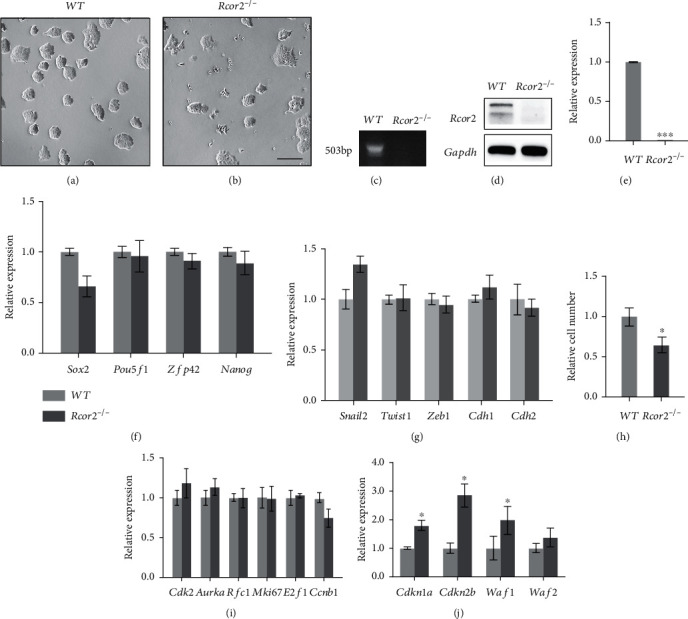
*Rcor2* is required for mouse embryonic stem cell proliferation. (a, b) The colonies of wild type (a) and *Rcor2^−/−^* (b) mESCs cultured in 2i medium. Scale bars, 100 *μ*m. (c) Genotyping of WT mESCs and *Rcor2^−/−^* mESCs. (d) Western blot of Rcor2 protein level in WT mESCs and *Rcor2^−/−^* mESCs. (e) Relative expression of *Rcor2* in WT mESCs and *Rcor2^−/−^* mESCs. (f, g) Relative expression of pluripotent (f) and EMT (g) related genes in WT mESCs and *Rcor2^−/−^* mESCs. (h) Relative cell number of WT mESCs and *Rcor2^−/−^* mESCs after 3 days culture. (i, j) Relative expression of cell cycle-related genes which promote proliferation (i) and inhibit proliferation (j) in WT mESCs and *Rcor2^−/−^* mESCs. Data are expressed as mean ± SEM, *n* = 3, ^∗^*p* < 0.05, ^∗∗^*p* < 0.01, ^∗∗∗^*p* < 0.001.

To investigate the function of *Rcor2* in mESC differentiations, we generated a *Rcor2^−/−^* mESC cell line using CRISPR/Cas9 system, with the fifth and the sixth exons of *Rcor2* knocked out in this cell line. The *Rcor2*^−/−^ mESC can be maintained in 2i medium. Wild-type (WT) mESCs form well-defined colonies (Figure 1(a)), while the colonies of *Rcor2^−/−^* mESCs appear less well defined and often spread and merge into irregular colonies (Figure 1(b)). Genotyping (PCR on genomic DNA from exon5 to exon7), qRT-PCR analysis, and western blot confirmed the knockout of *Rcor2* gene and the absence of *Rcor2* expression in *Rcor2^−/−^* mESCs (Figures 1(c)–1(e)), and no significant differences of the expression of pluripotent genes were observed between WT and *Rcor2^−/−^* mESCs, except slightly reduced *Sox2* expression in *Rcor2^−/−^* mESCs (Figure 1(f)). To investigate whether the irregular morphology of *Rcor2^−/−^* mESC colonies is related to epithelial-mesenchymal transition (EMT), an early step in the differentiation of ESCs, we examined the expression level of EMT marker genes, and no differences were found in *Snail2*, *Twist1*, *Zeb1*, *Cdh1*, and *Cdh2* expression between *Rcor2^−/−^* and WT mESCs (Figure 1(g)), indicating that *Rcor2* does not participate in EMT regulation in mESCs. It has been reported that the proliferation of mESCs with *Rcor2* knockdown is slower than the WT mESCs [10]. Therefore, we examined cell proliferation in *Rcor2^−/−^* and WT mESCs by counting the cell numbers after 3 days of culture started with the same cell number. We found that the cells in *Rcor2^−/−^* mESCs were significantly fewer than WT mESCs (Figure 1(h)), confirming that cell proliferation is impaired by *Rcor2* knockout. Next, we examined the cell cycle-related genes in these two mESC lines and found that the genes encoding proteins promoting cell proliferation were not significantly changed in *Rcor2^−/−^* mESCs (Figure 1(i)), while the genes encoding proteins that inhibit cell cycle, such as *Cdkn1a* and *Cdkn2b*, were significantly increased in *Rcor2^−/−^* mESCs compared to WT mESCs (Figure 1(j)), indicating Rcor2 promotes mESC proliferation by inhibiting expression of genes negatively regulating cell cycle. In summary, *Rcor2*^*-/*-^ mESCs showed no difference of pluripotency and EMT compared to WT mESCs. However, *Rcor2^−/−^* mESCs are less proliferative than WT mESCs with increased expression of genes encoding proliferation inhibitors.

### 3.2. *Rcor2* Is Required for Differentiation of All Three Germ Layers

**Figure 2:**
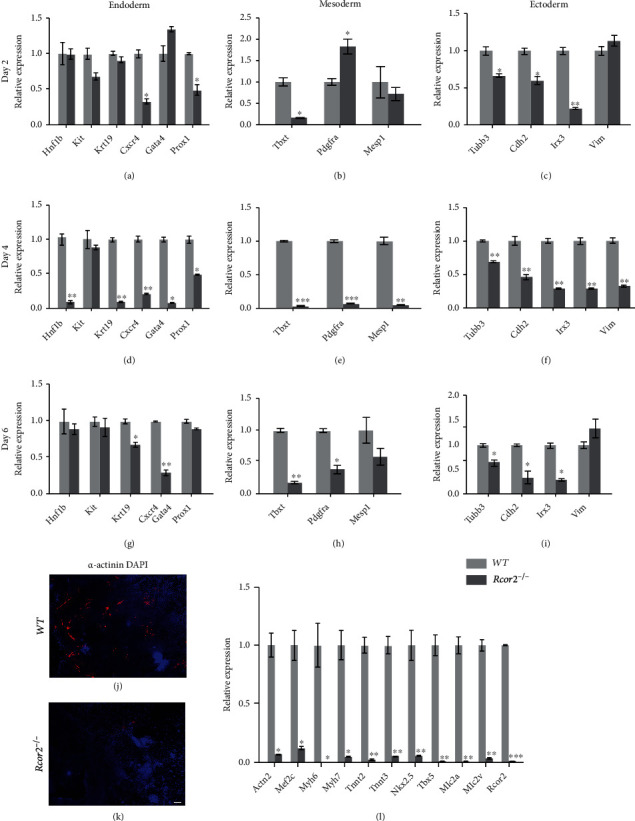
*Rcor2* is required for differentiation of all three germ layers. (a–i) Relative expression of endoderm (a, d, g), mesoderm (b, e, h), and ectoderm (c, f, i) specific genes in WT mESC-derived cells and *Rcor2*^*-/*-^ mESC-derived cells at day 2 (a–c), day 4 (d–f), and day 6 (g–i) of spontaneous random differentiation. (j, k) Immunofluorescence staining for DAPI (blue) and *α*-actinin (red) in WT (j) and *Rcor2^−/−^* (k) mESC-derived cells at day 9 of differentiation. Scale bars, 100 *μ*m. (l) Relative expression of cardiomyocyte maker genes in WT mESC-derived cells and *Rcor2^−/−^* mESC-derived cells at day 9 of differentiation. Data are expressed as mean ± SEM, *n* = 3, ^∗^*p* < 0.05, ^∗∗^*p* < 0.01, ^∗∗∗^*p* < 0.001.

To examine whether *Rcor2* is required for differentiation of mESCs, we induced spontaneous random differentiation of the *Rcor2^−/−^* and WT mESCs through embryoid bodies (EB). EBs were collected at day 2, day 4, and day 6 of differentiation, and qRT-PCR showed that several key marker genes of endoderm (*Cxcr4*), mesoderm (*Tbxt*), and ectoderm (*Irx3*) were significantly reduced in day 2 EBs from *Rcor2^−/−^* mESCs compared to those from WT mESCs (Figures 2(a)–2(c)). Most of the marker genes of the three germ layers were significantly reduced, if not diminished, in day 4 EBs (Figures 2(d)–2(f)) and remain reduced in day 6 EBs from *Rcor2^−/−^* mESCs compared to those from WT mESCs (Figures 2(g)–2(i)). These results suggested that *Rcor2* is required for differentiation of all three germ layers. In addition, we also tested the expression of inhibitors of cell cycle, which were elevated in *Rcor2^−/−^* mESCs (Figure 1(j)), during differentiation, and we found that most of the cell cycle inhibitors are upregulated at day 0 and day 2 of differentiation in *Rcor2*^−/−^ mESCs, consistent with undifferentiated mESCs (Figure 1(j)), while the changes of their expression are mixed at day 4 and day 9 of differentiation (Supplementary Fig. 1(a)-(d)), suggesting cell cycle is also dysregulated during differentiation of *Rcor2*^−/−^ cells. We then sought to determine whether *Rcor2^−/−^* mESCs can differentiate into terminally differentiated cell types, such as cardiomyocytes. We induced directed differentiation toward cardiomyocytes in both WT and *Rcor2^−/−^* mESCs. Immunofluorescent staining of *α*-actinin, a marker of cardiomyocytes, at day 9 of differentiation, showed WT mESCs differentiated to abundant *α*-actinin positive cardiomyocytes while *α*-actinin positive cells are strikingly absent in the *Rcor2^−/−^* cells (Figures 2(j) and 2(k)). qRT-PCR analysis also confirmed that the expression of cardiomyocyte-specific genes was drastically decreased in *Rcor2^−/−^* mESC-derived cells compared to WT mESC-derived cells (Figure 2(l)). It is well known that *Rcor2* was required for neurogenesis [8], and our results showed that *Rcor2* has an important role in differentiation of all three germ layers.

### 3.3. Absence of *Rcor2* Promotes Expression of Primordial Germ Cell Specific Genes

**Figure 3:**
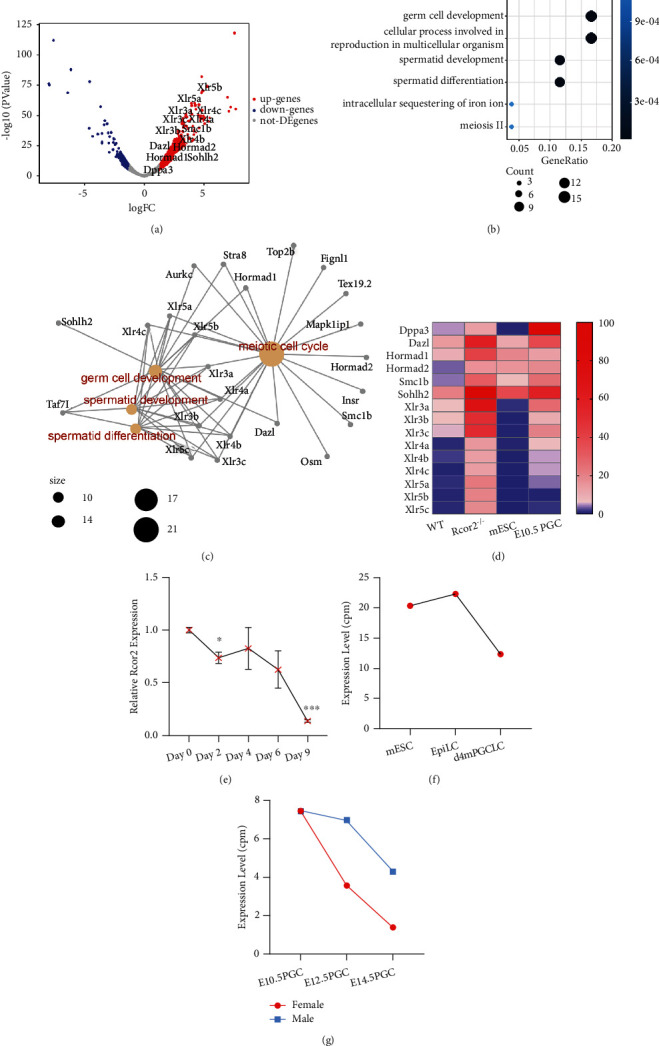
RNA-Seq analysis reveals enhanced germ cell specific gene expression in *Rcor2^−/−^* mESCs. (a) Volcano plot of gene expression changes in *Rcor2^−/−^* mESCs compared to WT mESCs. The blue, red, and gray dots indicate genes downregulated, upregulated, and unchanged, respectively (threshold = 2 folds change, Log2FC = 1). (b) GO term analysis of biological processes enriched from the upregulated genes in *Rcor2^−/−^* mESCs (no biological processes are enriched in downregulated genes). (c) The relationship of the upregulated genes in *Rcor2^−/−^* mESCs (black) and the enriched biological processes (red). (d) Heat map of PGC-related upregulated genes in *Rcor2^−/−^* mESCs and in WT and *Rcor2^−/−^* mESCs, WT mESCs, and E10.5 PGC [13]. (e) The relative expression of Rcor2 in WT mESCs at day 0, day 2, day 6, and day 9 of spontaneous random differentiation. (f) The count per million (cpm) of Rcor2 from exiting RNA-Seq datasets during PGC-like cell (PGCLC) differentiation [14]. (g) The expression of Rcor2 in count per million (cpm) from published RNA-Seq datasets during E10.5 PGC stage to E14.5 PGC stage [13]. (e) Data are expressed as mean ± SEM, *n* = 3, ^∗^*p* < 0.05, ^∗∗^*p* < 0.01, ^∗∗∗^*p* < 0.001.

To investigate the molecular mechanism of how Rcor2 affects differentiation of germ layers, the *Rcor2^−/−^* and WT mESCs were harvested for RNA-seq before spontaneous random differentiation. There were significantly more genes upregulated (302) in *Rcor2^−/−^* mESCs than the downregulated genes (123) compared to WT mESCs (Figure 3(a)), consistent with the known function of Rcor2 as a transcription suppressor [4]. GO term analysis showed that these upregulated genes were significantly enriched in germ cell development and spermatid development and differentiation (Figures 3(b) and 3(c)), suggesting that Rcor2 specifically regulates processes related to germ cells. Primordial germ cells (PGCs) are the precursors of sperm cells and eggs, and it is known that mouse PGCs are derived from within the posterior epiblast [12], in parallel with the three germ layers. We examined the expression level of the upregulated genes in *Rcor2^−/−^* mESCs in existing RNA-Seq datasets of mESC and E10.5 PGCs [13]. As expected, the expression level of upregulated genes related to germ cell development in *Rcor2^−/−^* mESCs was significantly higher in E10.5 PGCs compared to undifferentiated mESCs (Figure 3(d)), confirming the relevance of the upregulated genes in *Rcor2^−/−^* mESCs to the PGCs. Reversely, we found that the expression of *Rcor2* decreased along our spontaneous differentiation of WT mESCs (Figure 3(e)), and we also found that *Rcor2* expression decreased during PGC-like cell (PGCLC) differentiation from mESCs, by analyzing published RNA-Seq datasets (Figure 3(f)) [14]. In consistence with these data, we found that the expression level of *Rcor2* is significantly reduced from E10.5 PGCs to E14.5 PGCs *in vivo*, by analyzing published RNA-Seq datasets (Figure 3(g)) [13]. Upregulation of PGC-specific genes in *Rcor2^−/−^* mESCs, together with downregulation of Rcor2 in PGC differentiation, strongly suggests that Rcor2 plays an important role in suppressing PGC differentiation.

**Figure 4:**
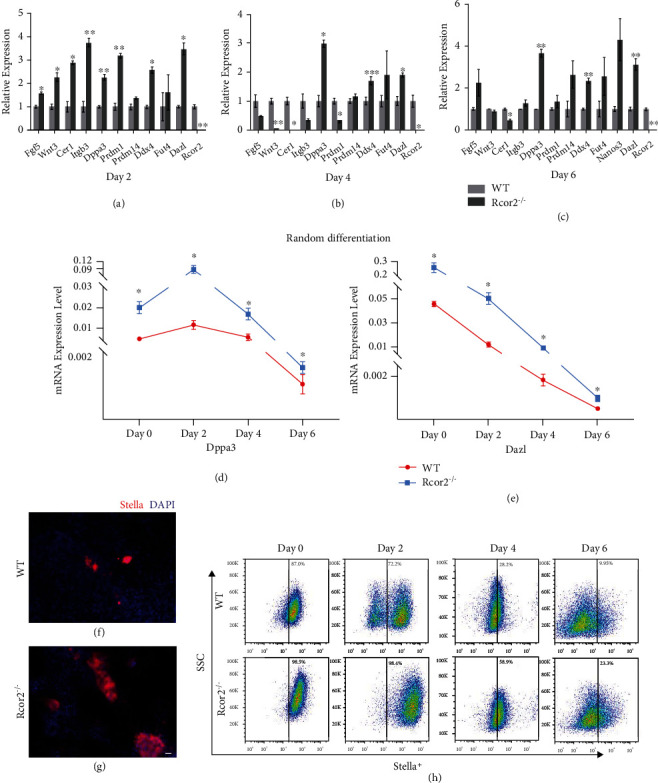
*Rcor2* knockout promotes PGC differentiation. (a–c) Relative expression of PGC marker genes in WT and *Rcor2^−/−^* mESC-derived cells at day 2 (a), day 4 (b), and day 6 (c) of spontaneous random differentiation. (d, e) mRNA expression level of *Dppa3* (d) and *Dazl* (e) during spontaneous random differentiation at day 0, day 2, day 4, and day 6. (f, g) Immunofluorescence staining for DAPI (blue) and Stella (red) in WT (f) and *Rcor2^−/−^* (g) mESC-derived cells at day 9 of spontaneous random differentiation. Scale bars, 100 *μ*m. (h) Flow cytometric analysis of Stella positive cell in WT and *Rcor2^−/−^* mESC-derived cells during spontaneous random differentiation at day 0, day 2, day 4, and day 6. Data are expressed as mean ± SEM, *n* = 3, ^∗^*p* < 0.05, ^∗∗^*p* < 0.01, ^∗∗∗^*p* < 0.001.

To investigate whether PGC differentiation is altered in *Rcor2^−/−^* mESCs, spontaneous random differentiation was conducted in *Rcor2^−/−^* and WT mESCs. It has been reported primordial germ cell-like cells (PGCLCs) were generated from mESCs through epiblast-like cells (EpiLCs) [15]. We examined the expression level of epiblast markers such as *Fgf5* and *Wnt3* and genes specifically upregulated upon PGC specification such as *Dppa3* and *Dazl* at day 2, day 4, day 6, and day 9 of differentiation. Epiblast marker genes were significantly elevated at day 2 of differentiation in *Rcor2^−/−^* mESCs compared to WT mESC, then gradually decreased at day 4, day 6, and day 9 of differentiation in *Rcor2^−/−^* mESC-derived cells. However, the majority of PGC marker genes, especially *Dppa3* and *Dazl*, were significantly upregulated in *Rcor2^−/−^* mESC-derived cells throughout the differentiation compared to WT mESC-derived cells (Figures 4(a)–4(c), Supplementary Fig. 2). *Dppa3* is a PGC-specific gene involved in epigenetic chromatin reprogramming [16]. *Dazl* is essential for gametogenesis in both males and females, playing a major role during spermatogenesis [17]. The dynamics of the expression of *Dppa3* and *Dazl* during spontaneous random differentiation showed that the expression of these two genes was always higher in *Rcor2^−/−^* mESC-derived cells than WT mESC-derived cells at all-time points examined (Figures 4(d) and 4(e)), with the expression of *Dppa3* peaked at day 2, then gradually decreased during the differentiation, while the expression of *Dazl* showed a downward trend throughout the differentiation.

We stained Stella, which is the protein coded by *Dppa3*, at day 9 of differentiation (Figures 4(f) and 4(g)), and found that the Stella positive cells in WT cells were significantly fewer than in the *Rcor2^−/−^* cells. Flow cytometry using Stella antibody showed that the percentage of Stella positive cells of *Rcor2^−/−^* mESC-derived cells was consistently higher than WT mESC-derived cells at day 2, day 4, and day 6 of differentiation (Figure 4(h)). Combined with the qRT-PCR results (Figures 4(a)–4(d)), these results showed that Rcor2 suppresses expression of *Dppa3* and other genes which upregulated in PGC and inhibits PGC differentiation. In summary, we found that *Rcor2^−/−^* mESCs had limited capacity to differentiate to all three germ layers, but it showed a surprisingly enhanced capacity to differentiate to PGCs, suggesting that Rcor2 may function in somatic cell fate determination by suppressing PGC fate during mESC differentiation.

### 3.4. *Rcor2* Inhibits *Dppa3* and *Dazl* Expression by Binding to Their Promoters

**Figure 5:**
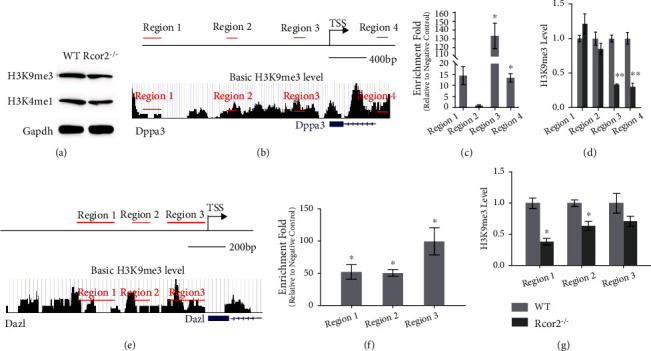
*Rcor2* inhibits *Dppa3* and *Dazl* expression by enhancing H3K9me3 on their promoters. (a) The protein level of H3K4me1 and H3K9me3 in WT and *Rcor2^−/−^* mESCs. (b, e) Diagrams of the tested regions by ChIP-qPCR on *Dppa3* (b) and *Dazl* (e) genes and basal H3K9me3 level on *Dppa3* (b) and *Dazl* (e) genes in mESCs (data from GEO: GSM32218, generated by ENCODE [27]). (c, f) ChIP-qPCR shows the enrichment of Rcor2 on *Dppa3* (c) and *Dazl* (f) promoters at the tested regions, relative to their respective negative control regions, which are located in gene bodies (not shown). Data are expressed as mean ± SEM, *n* = 3, ^∗^*p* < 0.05. (d, g) ChIP-qPCR shows the H3K9me3 level at the tested regions of *Dppa3* (d) and *Dazl* (g) genes in WT and *Rcor2^−/−^* mESCs. Data are expressed as mean ± SEM, *n* = 3, ^∗^*p* < 0.05, ^∗∗^*p* < 0.01, ^∗∗∗^*p* < 0.001.

Various observations showed that DNA and histone demethylation play an important role during germ cell development to activate the germ cell-related genes and inactivate genes of the somatic fate [18]. Rcor2 is known to be a suppressor of gene expression, through forming complexes with other suppressive proteins such as REST and LSD1 on the promoters of targeted genes [4, 10]. And it has been reported the genes occupied by Rcor2 and LSD1 were with significantly reduced histone H3 lysine 4 (H3K4me1), which is linked to active transcription. In addition, pilocarpine-induced seizures are associated with increased protein levels of LSD1 and Rcor2 in accordance with increased H3 lysine 9 trimethylation (H3K9me3), which is generally associated with silenced genes [19, 20]. Thus, we examined the protein level of H3K9me3 and H3K4me1 in *Rcor2^−/−^* mESCs and WT mESCs, and our results showed a slight reduction of both H3K9me3 and H3K4me1 in *Rcor2^−/−^* mESCs compared to WT mESCs (Figure 5(a)). The decreased H3K9me3 is in accordance with our RNA-Seq data which showed more upregulated genes (including germ cell-related genes) compared to downregulated genes upon *Rcor2* deletion (Figures 3(a)–3(d)). What is more, it was also found that H3K9me3 is exclusively reduced in the germ cell at E11.5 rather than in the somatic nuclei [21]. Thus, H3K9me3 modification may likely mediate the suppression of germ cell-related genes by Rcor2. Firstly, to test whether Rcor2 is associated with the promoter of *Dppa3* and *Dazl* genes, we utilized the *Rcor2^Flag^* mESC, in which 3 × Flag sequences were inserted behind the start codon of *Rcor2* gene by CRISPR/Cas9 system. The *Rcor2^Flag^* mESCs cultured in ES medium (subtracting 2i from 2i medium) were collected, and then, Chromatin IP was performed using Flag antibody. Association of Rcor2 with regions of *Dppa3* and *Dazl* genes was tested by Chromatin IP qPCR (Figures 5(b) and 5(e)), which showed enrichment of Rcor2 binding in regions surrounding the TSS of *Dppa3* and *Dazl* (Figures 5(c) and 5(f)). Given the binding of Rcor2 on the *Dppa3* and *Dazl* genes and decreased H3K9me3 modification in *Rcor2^−/−^* mESCs, we hypothesized that *Rcor2* can regulate the H3K9me3 modification on *Dppa3* and *Dazl* promoters. ChIP-qPCR using antibody against H3K9me3 was performed to measure the H3K9me3 level on *Dppa3* and *Dazl* promoters, in mESCs harvested at day 2 of spontaneous random differentiation. In the *Rcor2* binding regions of *Dppa3* and *Dazl*, the H3K9me3 level significantly decreased in the *Rcor2^−/−^* mESC-derived cells compared to WT mESC-derived cells (Figures 5(d) and 5(g)). These results showed that, in the absence of *Rcor2*, H3K9me3 level on the promoters of *Dppa3* and *Dazl* genes decreased, thus likely allowing activation of expression of both genes. These results suggested that Rcor2 suppresses the expression of *Dppa3* and *Dazl* by mediating H3K9me3 modification on their promoters.

## 4. Discussion

We utilized a *Rcor2^−/−^* mESC cell line to investigate the role of *Rcor2* in ESC differentiation and found that *Rcor2^−/−^* mESCs have drastically diminished capacity to differentiate into all three germ layers. Intriguingly, PGC differentiation was enhanced in *Rcor2^−/−^* mESCs, and we discovered that Rcor2 binds to promoters of genes important for PGC fate determination, such as *Dppa3* and *Dazl*, and mediates their H3K9me3 modification, thus suppressing their expression and inhibiting PGC differentiation. Therefore, we revealed a novel function of Rcor2 on suppressing germ cell fate to allow differentiation of the somatic lineages.

Unlike in some animals that the germ cells are predetermined in early development, mouse germ cells' fate is induced in postimplantation epiblasts [22]. Thus, it is critical to properly segregate the fates of PGCs and the somatic cells in the surrounding germ layers receiving similar inductive signals. After specification at embryonic days 6.25-7.25, mouse PGCs undergo a series of reprogramming events to repress their somatic fate, including DNA methylation and histone modification [23]. However, it is less known how somatic cells solidify their germ layer fate against the germ line fate. Our finding that Rcor2 can function to specifically suppress expression of PGC genes offers novel insight into the mechanism how somatic cells guard their identity and fate. It remains an interesting question how this specificity is achieved. Interaction between Rcor2 and transcription factors specifically binding to promoters of PGC genes may be a possible molecular mechanism.

Rcor2 is known to be recruited by REST [4] to regulate cell fate decision during neural differentiation and has also been shown to be associated with LSD1 to regulate neural development [10]. LSD1 demethylates either dimethylated or monomethylated lysine 4 residues on histone 3 (H3K4), and it has also been reported that LSD1 demethylates the histone 3 at lysine 9 (H3K9) [24]. Multiple studies had indicated that coRESTs play a crucial role in H3K4 demethylation [6, 8]. However, little is known about the function of coREST on H3K9me3 modification. H3K9me3 has been reported to play an essential role during cell fate determination, and removing H3K9me3 could enhance the efficiency of reprogramming [25]. Our results shows that Rcor2 can regulate H3K9me3 modification on the promoters of its PGC-specific targets such as *Dppa3* and *Dazl*, suggesting coRESTs also regulate H3K9me3 in the context of cell fate determination between PGCs and germ layers. Further investigations are warranted to examine whether LSD1 is involved in this process, and whether PGC-specific transcription factors are required.

Furthermore, various studies have demonstrated that PGC-like cells could be generated from ESCs, and about 40%-60% cells could become PGCs at day 6 of differentiation [15]. It also has been showed that much fewer cells can develop into viable sperm and oocytes *in vitro* [26]. Our study found the repressive role of *Rcor2* in germ cell differentiation, which may provide insights and possible targets to develop novel methods with enhanced efficiency of germ cell reconstitution and gametogenesis from mESCs.

## Data Availability

The data that support the findings of this study are available from the corresponding author upon reasonable request.

## Abbreviations

ESC: Embryonic stem cells
mESC: Mouse embryonic stem cell
PGCs: Promotes primordial germ cells
REST: RE-1 silencing transcription factor
LSD1: Lysine demethylase 1A
WT: Wild type
EMT: Epithelial-mesenchymal transition
EB: Embryoid bodies
DAPI: 4′,6-Diamidino-2-phenylindole
PGCLCs: Primordial germ cell-like cells
EpiLCs: Epiblast-like cells.

## Supplementary Materials

Supplementary Materials Supplementary Figure 1: the cell cycle-related gene expression during differentiation. (a–d) Relative expression of cell cycle-related genes in WT mESC-derived cells and *Rcor2*^*-/*-^ mESC-derived cells at day 0 (a), day 2 (b), day 6 (c), and day 9 (d) of differentiation. Supplementary Figure 2: *Rcor2* knockout promotes PGC differentiation. (a) Relative expression of PGC marker genes in WT and *Rcor2*^−/−^ mESC-derived cells at day 9 of differentiation. Supplementary Table 1: sequences of primers used.
