# Supplementary material for: Rcor2 Is Required for Somatic Differentiation and Represses Germline Cell Fate
Source: Stem Cells Int. 2022 Mar 19;2022:5283615. doi: 10.1155/2022/5283615 (PMC8957467; doi:10.1155/2022/5283615)
Supplement: Supplementary Materials — Supplementary Figure 1: the cell cycle-related gene expression during differentiation. (a–d) Relative expression of cell cycle-related genes in WT mESC-derived cells and Rcor2-/- mESC-derived cells at day 0 (a), day 2 (b), day 6 (c), and day 9 (d) of differentiation. Supplementary Figure 2: Rcor2 knockout promotes PGC differentiation. (a) Relative expression of PGC marker genes in WT and Rcor2−/− mESC-derived cells at day 9 of differentiation. Supplementary Table 1: sequences of primers used. [file 5283615.f1.docx]

**Supplementary Material for**

**Rcor2 is required for somatic differentiation and represses germline cell fate**

**Figure Legends**

**Supplementary Figure 1. The cell cycle related gene expression during differentiation.**

(a-d) Relative expression of cell cycle related genes in WT mESCs derived cells and *Rcor2^-/^*^-^ mESCs derived cells at day 0 (a), day 2 (b), day 6 (c) and day 9 (d) of differentiation.

Data are expressed as mean ± SEM, n=3, *p<0.05, **p<0.01, ***p<0.001.

**Supplementary Figure 2. *Rcor2* knockout promotes PGC differentiation.**

(a) Relative expression of PGC marker genes in WT and *Rcor2*^-/-^ mESCs derived cells at day 9 of differentiation.

Data are expressed as mean ± SEM, n=3, *p<0.05, **p<0.01, ***p<0.001.

**Supplementary Figure 1. The cell cycle related gene expression during differentiation.**


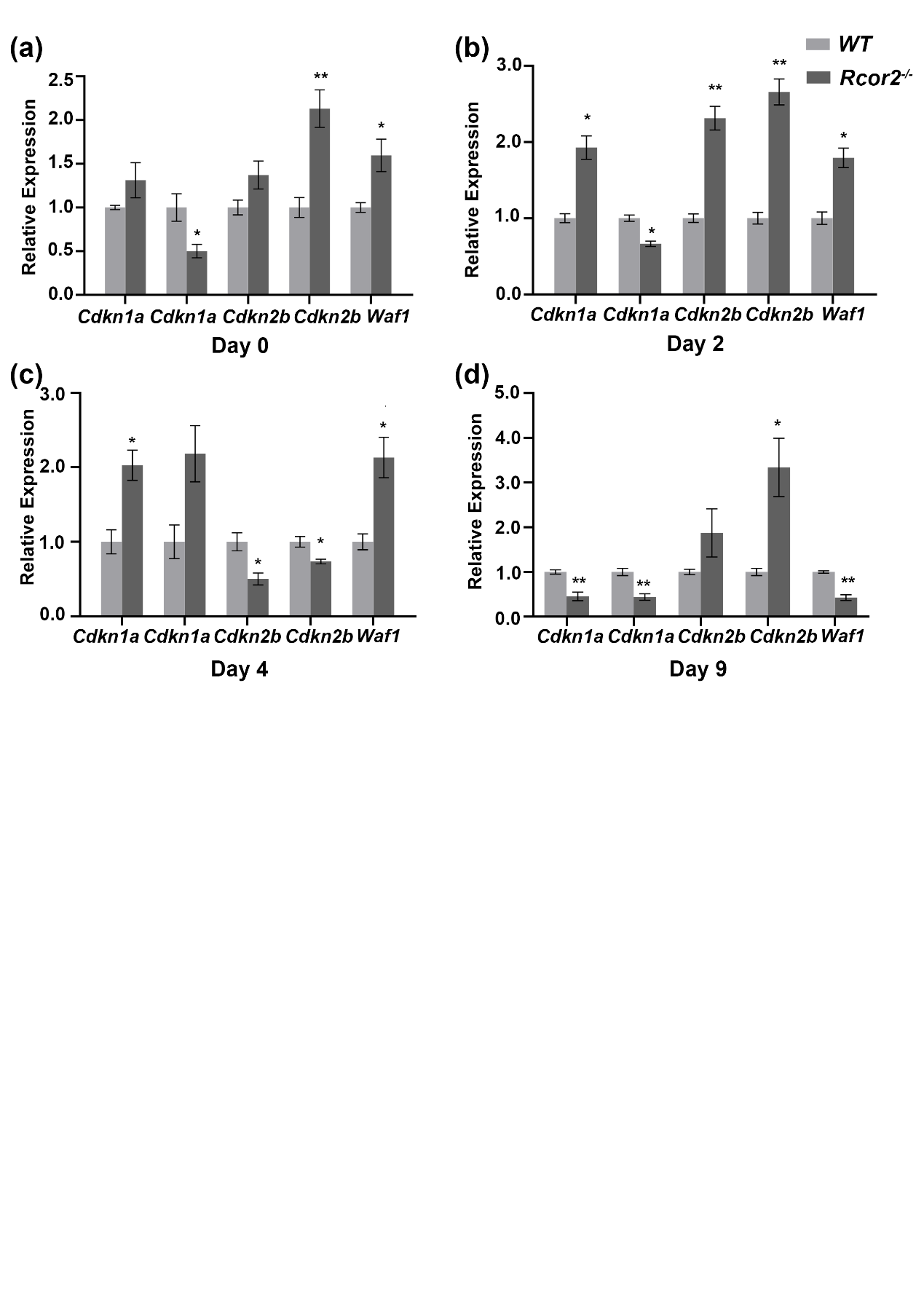


**Supplementary Figure 2. *Rcor2* knockout promotes PGC differentiation.**


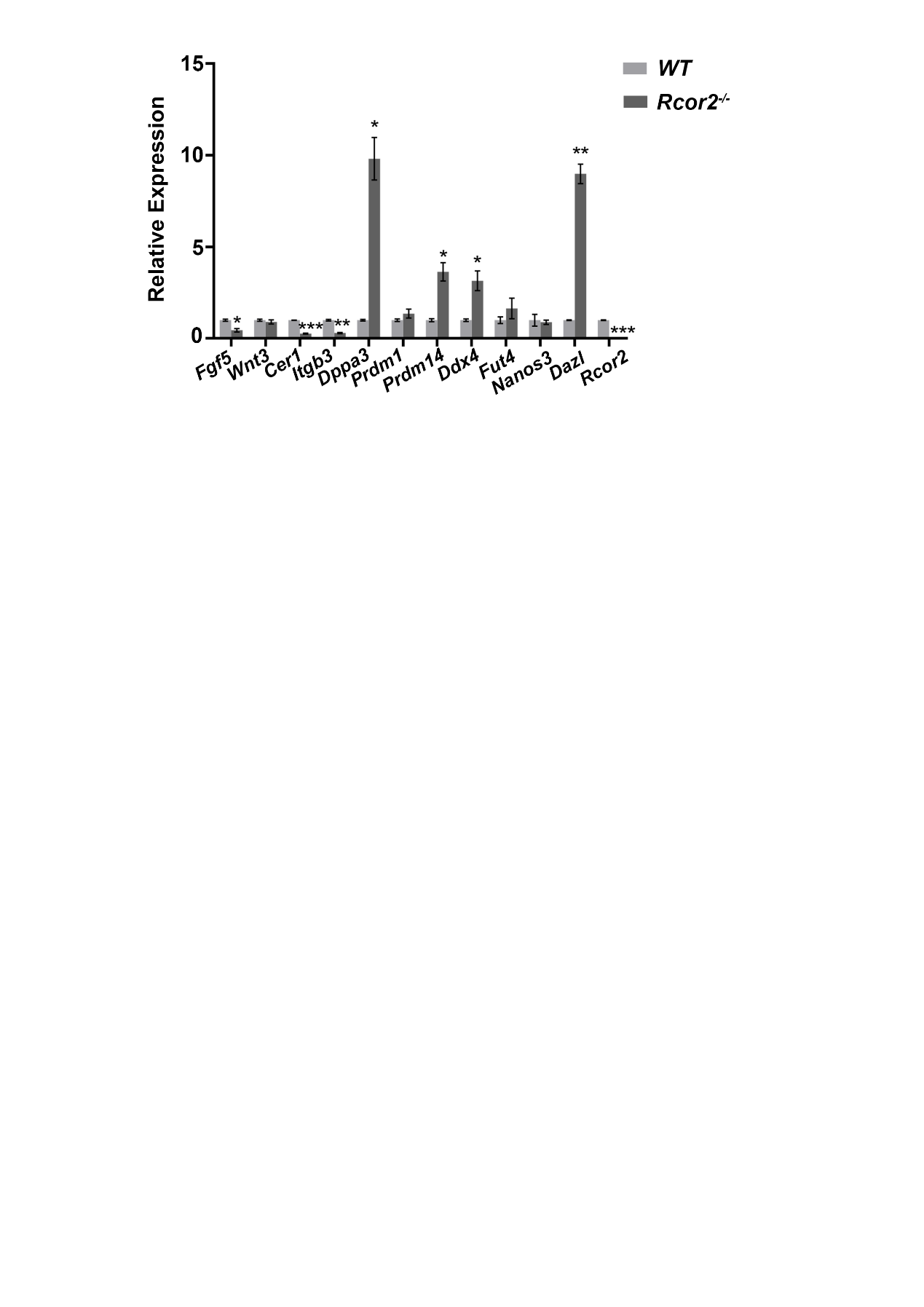


**Supplementary Table 1. Sequences of primers used**

| Gene | 5’-3’ |
| --- | --- |
| Rcor2-F | TAGTGTGATGGACAGGCAGGC |
| Rcor2-R | GCTTGGGGTCTCCAGTATCCG |
| Sox2-F | GCGGAGTGGAAACTTTTGTCC |
| Sox2-R | CGGGAAGCGTGTACTTATCCTT |
| Pou5f1-F | GGCTTCAGACTTCGCCTCC |
| Pou5f1-R | AACCTGAGGTCCACAGTATGC |
| Nanog-F | TCTTCCTGGTCCCCACAGTTT |
| Nanog-R | GCAAGAATAGTTCTCGGGATGAA |
| Zfp42-F | CCCTCGACAGACTGACCCTAA |
| Zfp42-R | TCGGGGCTAATCTCACTTTCAT |
| Gapdh-F | AGGTCGGTGTGAACGGATTTG |
| Gapdh-R | TGTAGACCATGTAGTTGAGGTCA |
| Fgf5-F | TGTGTCTCAGGGGATTGTAGG |
| Fgf5-R | AGCTGTTTTCTTGGAATCTCTCC |
| Wnt3-F | CTCGCTGGCTACCCAATTTG |
| Wnt3-R | CTTCACACCTTCTGCTACGCT |
| Cerl-F | CTCTGGGGAAGGCAGACCTAT |
| Cerl-R | CCACAAACAGATCCGGCTT |
| Itgb3-F | CCACACGAGGCGTGAACTC |
| Itgb3-R | CTTCAGGTTACATCGGGGTGA |
| Dppa3-F | GACCCAATGAAGGACCCTGAA |
| Dppa3-R | GCTTGACACCGGGGTTTAG |
| Prdm1-F | TTCTCTTGGAAAAACGTGTGGG |
| Prdm1-R | GGAGCCGGAGCTAGACTTG |
| Prdm14-F | ACTGCCTCCAGCATACTCCG |
| Prdm14-R | CCATTGTCCTTGTGTGAGCG |
| Ddx4-F | GCTTCATCAGATATTGGCGAGT |
| Ddx4-R | GCTTGGAAAACCCTCTGCTT |
| Fut4-F | TAAGGCGCTACCAGTGTTCG |
| Fut4-R | GGACGAGAACCTACCAGGG |
| Nanos3-F | TACACCACCCGGAATTCTGC |
| Nanos3-R | CCGTAGTGGAGGGACAGCAG |
| Dazl-F | ATGTCTGCCACAACTTCTGAG |
| Dazl-R | CTGATTTCGGTTTCATCCATCCT |
| Cdh1-F | CAGGTCTCCTCATGGCTTTGC |
| Cdh1-R | CTTCCGAAAAGAAGGCTGTCC |
| Snail-F | TGGTCAAGAAACATTTCAACGCC |
| Snail-R | GGTGAGGATCTCTGGTTTTGGTA |
| Zeb1-F | GCTGGCAAGACAACGTGAAAG |
| Zeb1-R | GCCTCAGGATAAATGACGGC |
| Twist1-F | GGACAAGCTGAGCAAGATTCA |
| Twist1-R | CGGAGAAGGCGTAGCTGAG |
| Dppa3-Region1-F | GCAGGTGGATTTCTGAGTTCG |
| Dppa3-Region1-R | CTCCTGCCTCTGCTTCAATGT |
| Dppa3-Region2-F | ACATTGGAGGCAGAGAAAGGAAGAG |
| Dppa3-Region2-R | CTAACTCTTTGTTTGAGACTCCGTG |
| Dppa3-Region3-F | GAGAGCGGGGAATCCTACAGT |
| Dppa3-Region3-R | TACAGCCGAGTCTACCCCCAG |
| Dppa3-Region4-F | TGGAGGACCAGGGAGTTTCA |
| Dppa3-Region4-R | ACAGTCTCAACTCCCTCCCG |
| Ccnb1-F | CTACCGCACAACGCACTTTCT |
| Ccnb1-R | GCTTCTTCCTCCACTTCCCCT |
| E2f1-F | GGTGGCTGCTGACTCACTCCT |
| E2f1-R | GGGTCAAGTCCCCAAAGTCAC |
| Mad2-F | GTGTGTGTGACGCTGTCCTCG |
| Mad2-R | TGGACATCTTGCTCATCTGCC |
| Rfc1-F | AAAGCAGCCTTCACAAGAGCC |
| Rfc1-R | AGGGCATCCTGCTCTTTCTCA |
| Waf1-F | CGTGGCCTTGTCGCTGTCTT |
| Waf1-R | CGGCGCTTGGAGTGATAGAAA |
| Cdh2-F | GGAGGAGAGGAAGACCAGGAC |
| Cdh2-R | TTGTGGCTCAGCGTGGATAGG |
| Cdk2-F | CCTGCTTATCAATGCAGAGGG |
| Cdk2-R | TGCGGGTCACCATTTCAGC |
| Cdkn1a-F | CCTGGTGATGTCCGACCTG |
| Cdkn1a-R | CCATGAGCGCATCGCAATC |
| Cdkn2b-F | CCCTGCCACCCTTACCAGA |
| Cdkn2b-R | CAGATACCTCGCAATGTCACG |
| Mki67-F | ATCATTGACCGCTCCTTTAGGT |
| Mki67-R | GCTCGCCTTGATGGTTCCT |
| Aurka-F | AGACAAAGCAAGTTCATCCTGG |
| Aurka-R | TGTTCCAAGGGGCGCATATTC |
| Dazl-Region3-F | TAGGGAGTGGCAGGAGTCGGT |
| Dazl-Region3-F | CTGGCCTCGTGACGTGCTACA |
| Dazl-Region2-F | TGAAAAAGAGGGTGAAGGGGC |
| Dazl-Region2-F | GGCGTTATCCCGTCAGCAAAG |
| Dazl-Region1-F | AGCTCGGCTCTGCTGTAGGTA |
| Dazl-Region1-F | ATCCGGTACAAAGGACACAGC |
| Kdr-F | TTTGGCAAATACAACCCTTCAGA |
| Kdr-R | TTTGGCAAATACAACCCTTCAGA |
| Mesp1-F | AGTCGCTCGGTCCCCGTTTA |
| Mesp1-R | GCTGCTGAAGAGCGGAGATGA |
| Hnf1b-F | AGGGAGGTGGTCGATGTCA |
| Hnf1b-R | TCTGGACTGTCTGGTTGAACT |
| Kit-F | CTCCCCCAACAGTGTATTCAC |
| Kit-R | TAGCCCGAAATCGCAAATCTT |
| krt19-F | GGGGGTTCAGTACGCATTGG |
| krt19-R | GAGGACGAGGTCACGAAGC |
| Cxcr4-F | GAAGTGGGGTCTGGAGACTAT |
| Cxcr4-R | TTGCCGACTATGCCAGTCAAG |
| Gata4-F | CCCTACCCAGCCTACATGG |
| Gata4-R | ACATATCGAGATTGGGGTGTCT |
| Prox1-F | AGAAGGGTTGACATTGGAGTGA |
| Prox1-R | TGCGTGTTGCACCACAGAATA |
| Tubb3-F | CTCGGAGCAGTTCACAGCCAT |
| Tubb3-R | TCATACATCTCCCCCTCCTCC |
| lmx1a-F | CCCTATGGTGCTGAACCTCTT |
| lmx1a-R | CAGATGGTCAATGGGGTTTCC |
| Irx3-F | AGCCGGAGAGTGGAACAGATC |
| Irx3-R | AAGACCAGAGCAGCGTCCAGA |
| vim-F | CGTCCACACGCACCTACAG |
| vim-R | GGGGGATGAGGAATAGAGGCT |
| Cdkn1b-F | TCAAACGTGAGAGTGTCTAACG |
| Cdkn1b-R | CCGGGCCGAAGAGATTTCTG |
| Cdkn2a-F | CGCAGGTTCTTGGTCACTGT |
| Cdkn2a-R | TGTTCACGAAAGCCAGAGCG |
| Rcor2-F1 | GCCAACTTCACTCCCTTCCCT |
| Rcor2-R1 | GCTTGGGGTCTCCAGTATCCG |
